# Down-Regulation of Astrocytic Kir4.1 Channels during the Audiogenic Epileptogenesis in *Leucine-Rich Glioma-Inactivated 1* (*Lgi1*) Mutant Rats

**DOI:** 10.3390/ijms20051013

**Published:** 2019-02-26

**Authors:** Masato Kinboshi, Saki Shimizu, Tomoji Mashimo, Tadao Serikawa, Hidefumi Ito, Akio Ikeda, Ryosuke Takahashi, Yukihiro Ohno

**Affiliations:** 1 Department of Pharmacology, Osaka University of Pharmaceutical Sciences, 4-20-1 Nasahara, Takatsuki, Osaka 569-1094, Japan; kinboshi@kuhp.kyoto-u.ac.jp (M.K.); s.shimizu@gly.oups.ac.jp (S.S.); serikawa@anim.med.kyoto-u.ac.jp (T.S.); 2 Institute of Experimental Animal Sciences, Graduate School of Medicine, Osaka University, 2-2 Yamadaoka, Suita, Osaka 565-0871, Japan; mashimo@iexas.med.osaka-u.ac.jp; 3 Department of Neurology, Wakayama Medical University, 811-1 Kimiidera, Wakayama 641-8509, Japan; itohid@kuhp.kyoto-u.ac.jp; 4 Department of Epilepsy, Movement Disorders and Physiology, Graduate School of Medicine, Kyoto University, 54 Kawaharacho, Shogoin, Sakyo-ku, Kyoto 606-8507, Japan; akio@kuhp.kyoto-u.ac.jp; 5 Department of Neurology, Graduate School of Medicine, Kyoto University, 54 Kawaharacho, Shogoin, Sakyo-ku, Kyoto 606-8507, Japan; ryosuket@kuhp.kyoto-u.ac.jp

**Keywords:** astrocytes, epilepsy, Kir4.1 channels, Lgi1, ADLTE, antiepileptics, valproic acid

## Abstract

The dysfunction of astrocytic inwardly rectifying potassium (Kir) 4.1 channels, which mediate the spatial potassium-buffering function of astrocytes, is known to be involved in the development of epilepsy. Here, we analyzed the Kir4.1 expressional changes in *Leucine-Rich Glioma-Inactivated 1* (*Lgi1*) mutant rats, which is a model of autosomal dominant lateral temporal lobe epilepsy in humans, to clarify the role of astrocytic Kir4.1 channels in Lgi1-related epileptogenesis. Priming acoustic stimulation (at postnatal day 16) conferred seizure susceptibility on *Lgi1* mutant rats, which evoked audiogenic seizures with test stimulation at eight weeks. In the seizure-susceptible *Lgi1* mutant rats (before test stimulation), astrocytic Kir4.1 expression was down-regulated region-specifically in the cerebral cortex, hippocampus, and amygdala. In addition, prophylactic treatments of *Lgi1* mutant rats with valproic acid (VPA, 30 mg/kg and 200 mg/kg) for two weeks prevented both the development of seizure susceptibility and the down-regulation of Kir4.1 expression in astrocytes. The present study demonstrated for the first time that the astrocytic Kir4.1 expression was reduced in the *Lgi1*-related seizure model, suggesting that the down-regulation of Kir4.1 channels in astrocytes is involved in audiogenic epileptogenesis caused by *Lgi1* mutation. In addition, VPA seemed to have a prophylactic effect on *Lgi1*-related seizures.

## 1. Introduction

Astrocytes are a major component of glial cells in the brain that form tripartite synapses in conjunction with neuronal components (presynaptic nerve terminal and postsynaptic neuronal membrane), and actively regulate the excitability and plasticity of neurons. They maintain extracellular ion homeostasis, metabolize neurotransmitters (e.g., glutamate and GABA), and secrete various neuroactive substances (e.g., gliotransmitters, neurotrophic factors, and cytokines) [1,2,3]. Specifically, the spatial potassium (K^+^) buffering by astrocytes’ functions is a clearance mechanism of excessive extracellular K^+^ secreted from excited neurons, and is essential for controlling neuronal excitability. If astrocytic K^+^ buffering is disrupted, it increases the extracellular levels of K^+^ and glutamate at the synapses, and causes the abnormal excitation of neurons [4,5,6,7].

Spatial K^+^ buffering currents in astrocytes are primarily conducted by the inwardly rectifying K^+^ (Kir) channels containing Kir4.1 subunits (Kir4.1 channels), which consist of Kir4.1 homotetramer and Kir4.1/5.1 heterotetramer channels [7,8]. Previous studies using conditional knockout mice have shown that the specific deletion of Kir4.1 in astrocytes depolarized their resting membrane potential and markedly reduced the transport capacity of K^+^ and glutamate into astrocytes, causing ataxia, epileptic symptoms, and early mortality [9]. These findings suggest that disruption of the spatial K^+^ buffering function of astrocytes is associated with the induction of motor disorders, including epilepsy. In fact, loss-of-function mutations in the human *KCNJ10* gene encoding Kir4.1 were reported to cause the epileptic disorders known as “EAST” (Epilepsy, Ataxia, Sensorineural deafness, and Tubulopathy) or “SeSAME” (Seizures, Sensorineural deafness, Ataxia, Mental retardation, and Electrolyte imbalance) syndrome [10,11,12]. In addition, astrocytic Kir4.1 expression was known to be reduced (down-regulated) in some brain regions (e.g., hippocampus and amygdala) in rodent epilepsy models [13,14], and in seizure focus specimens from temporal lobe epilepsy (TLE) patients [15,16,17], suggesting that the reduced activity of the Kir4.1 channels evokes seizures. Furthermore, we previously showed that the inhibition (knockdown or blockade) of Kir4.1 channels facilitated the expression of brain-derived neurotrophic factor (BDNF) in astrocytes, which has long been implicated in the development of epilepsy (epileptogenesis) [18,19]. Interestingly, repeated treatments with antiepileptic drugs such as valproic acid (VPA) were found to up-regulate (elevate) the expression of astrocytic Kir4.1 channels in the limbic regions (e.g., amygdala and hippocampus) [20]. Therefore, astrocytic Kir4.1 channels seem to closely participate in the pathogenesis and treatment of epilepsy both in animals and in patients.

Autosomal dominant lateral temporal lobe epilepsy (ADLTE, OMIM 600512) is an epilepsy disorder with characteristic manifestations such as auditory auras and seizures triggered by auditory stimuli. ADLTE was reported to be caused by several heterozygous mutations of *leucine-rich glioma-inactivated 1* (*LGI1*) [21,22]. Moreover, the secreted synaptic protein Lgi1 has been shown to regulate the function of α-amino-3-hydroxy-5-methyl-4-isoxazolepropionic acid (AMPA)-type glutamate receptors and potassium channels (e.g., Kv1.1 channels) by forming complexes with a disintegrin and metalloproteinase protein 22 (ADAM22) and ADAM23, which are members of the membrane-anchored protein family [23,24,25,26]. However, the pathophysiological mechanisms underlying Lgi1-related epileptogenesis remain unknown.

We previously developed a novel rat model of Lgi1-related seizures that carries a missense mutation (L385R) of the *Lgi1* gene [27]. The *Lgi1* mutant rats showed audiogenic seizure susceptibility, resembling the clinical features of ADLTE [27,28]. In the present study, we evaluated the Kir4.1 expressional changes in astrocytes during the development of audiogenic epilepsy in *Lgi1* mutant rats to clarify the potential involvement of astrocytic Kir4.1 channels in Lgi1-related epileptogenesis. Moreover, we also studied the prophylactic actions of VPA in Lgi1-related epileptogenesis, with a focus on its regulation of Kir4.1 channel expression.

## 2. Results

### 2.1. Audiogenic Seizure Induction

**Figure 1:**
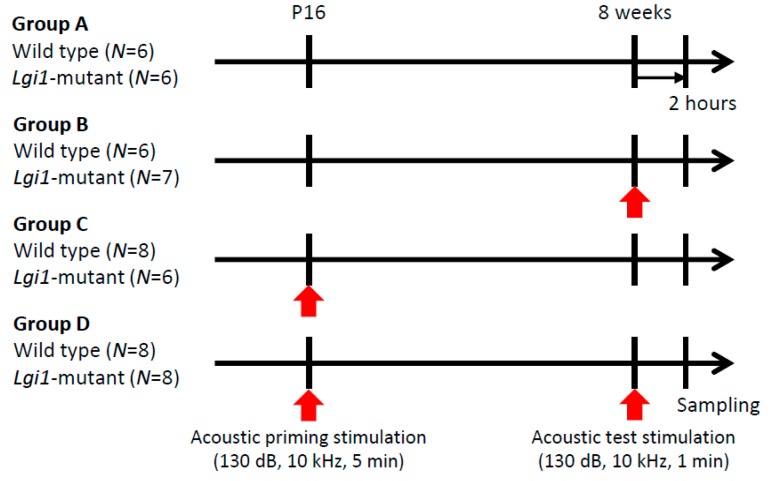
Audiogenic seizure induction. Wild-type rats and leucine-rich glioma-inactivated 1 (*Lgi1*) mutant rats were divided into four groups: Group A (no acoustic stimulation), Group B (only test stimulation), Group C (only priming stimulation), and Group D (both priming and test stimulation).

Wild-type (WT) rats and *Lgi1* mutant rats were divided into four groups. Groups A and B did not receive the acoustic priming stimulation without (Group A) and with (Group B) the test stimulation (130 dB, 10 kHz, one minute) at the age of eight weeks, respectively (Figure 1). Groups C and D received the acoustic priming stimulation (130 dB, 10 kHz, five minutes) at postnatal day (P) 16 without (Group C) and with (Group D) the test stimulation at eight weeks, respectively.

In all of the non-primed Group B animals (either WT or *Lgi1* mutant rats), the acoustic test stimulation at eight weeks did not cause any behavioral changes (Table 1). In contrast, in all of the primed Group D animals (either WT or *Lgi1* mutant rats), the acoustic test stimulation at eight weeks evoked wild running behavior. Moreover, *Lgi1* mutant rats (*n* = 8), except for one, showed generalized tonic–clonic seizures (GTCSs) following wild running, although none of the WT rats in Group D exhibited GTCSs (Table 1).

### 2.2. Expressional Changes in Astrocytic Kir4.1 during Audiogenic Epileptogenesis

**Figure 2:**
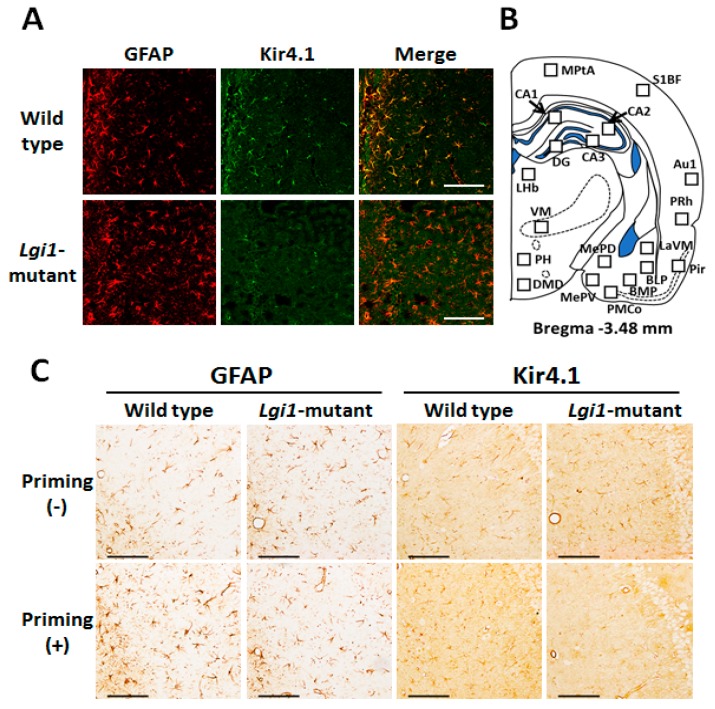
Kir4.1 expression in astrocytes. (**A**) Representative images of immunofluorescence double staining for glial fibrillary acidic protein (GFAP) and Kir4.1 in the hippocampal CA1 region in wild-type F344 rats (top panels) and *Lgi1* mutant rats (bottom panels). Scale bar: 100 μm. (**B**) Schematic illustration of a brain section (Bregma −3.48 mm level) selected for quantitative analysis of immunoreactivity (IR) of Kir4.1 or GFAP. Squares in each brain region indicate the areas analyzed for counting of Kir4.1-IR-positive or GFAP-IR-positive cells. Medial parietal association cortex (MPtA), primary somatosensory cortex barrel field (S1BF), primary auditory cortex (Au1), perirhinal cortex (PRh), piriform cortex (Pir), hippocampal CA1, CA2, CA3, and dentate gyrus (DG), medial amygdaloid nucleus posteroventral part (MePV), medial amygdaloid nucleus posterodorsal part (MePD), posteromedial cortical amygdaloid nucleus (PMCo), basomedial amygdaloid nucleus posterior part (BMP), basolateral amygdaloid nucleus posterior part (BLP), lateral amygdaloid nucleus ventromedial part (LaVM), lateral habenula (LHb), ventromedial thalamus (VM), posterior hypothalamus (PH), dorsomedial hypothalamic nucleus, and dorsal part (DMD). (**C**) Representative images of immunohistochemical staining for GFAP and Kir4.1 in the hippocampal CA1 regions of non-primed wild type rats (upper left panels), primed wild type rats (lower left panels), non-primed *Lgi1* mutant rats (upper right panels), and primed *Lgi1* mutant rats (lower right panels) after audiogenic seizure induction. Scale bar: 100 μm.

We first confirmed the expression pattern of Kir4.1 in WT F344 rats using the immunofluorescent double-staining techniques. As in other strains (Wistar and SD rats) [14,20,29], Kir4.1-immunoreactivity (IR) was mostly co-stained with glial fibrillary acidic protein (GFAP), which is a specific marker for astrocytes, in stellate-shaped cells (Figure 2A top), illustrating that Kir4.1 channels are specifically expressed in astrocytes in F344 rats. In addition, Kir4.1 expression in astrocytes was considerably reduced in the *Lgi1* mutant rats that received priming stimulation at P16 (Figure 2A bottom).

Topographical mapping analysis for the changes in Kir4.1 and GFAP expression was performed by counting the number of Kir4.1-IR-positive and GFAP-IR-positive cells stained by the avidin-biotin complex (ABC) method. Nineteen brain regions were analyzed, including the cerebral cortex, hippocampus, amygdala, thalamus, and hypothalamus, which were reported to be activated with audiogenic seizures in *Lgi1* mutant rats [28] (Figure 2B). Kir4.1-IR was mostly expressed in stellate-shaped cells, and there was no morphological difference in Kir4.1-IR or GFAP-IR positive cells between WT and *Lgi1* mutant rats (Figure 2C).

**Figure 3:**
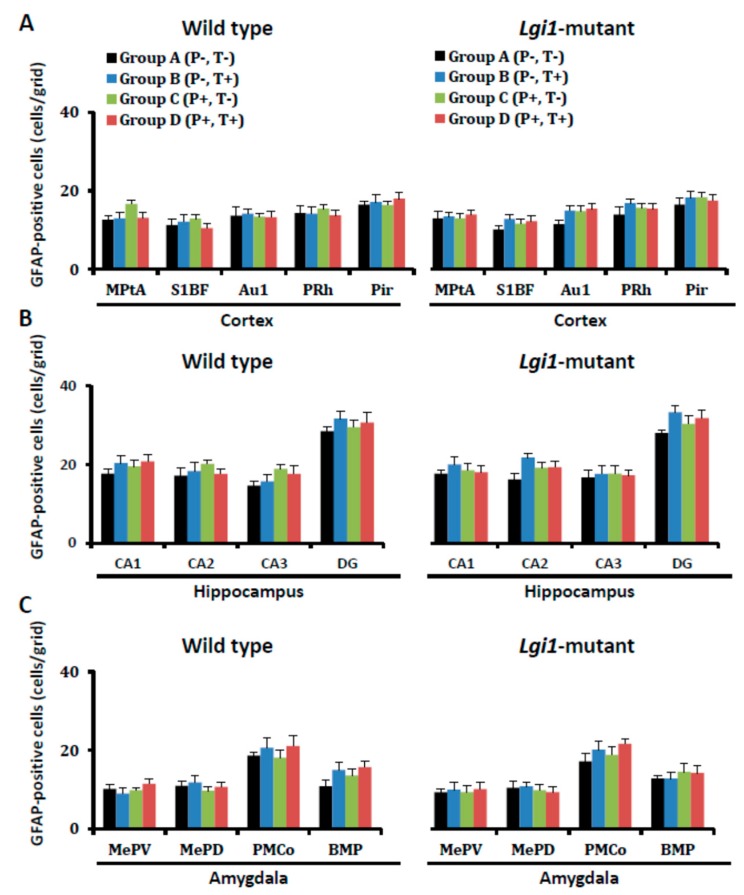
Changes in the number of GFAP-immunoreactivity (IR)-positive cells after development of audiogenic epilepsy. The GFAP-IR-positive cells in each group of wild-type rats (A–D) and *Lgi1* mutant rats are shown in each region of the cortex (**A**), hippocampus (**B**), and amygdala (**C**). Each point represents the mean ± S.E.M. of six to eight animals.

Neither priming stimulation at P16 nor test stimulation at eight weeks affected the number of astrocytes (GFAP-IR-positive cells) per se either in WT or *Lgi1* mutant rats of groups A–D (Figure 3).

**Figure 4:**
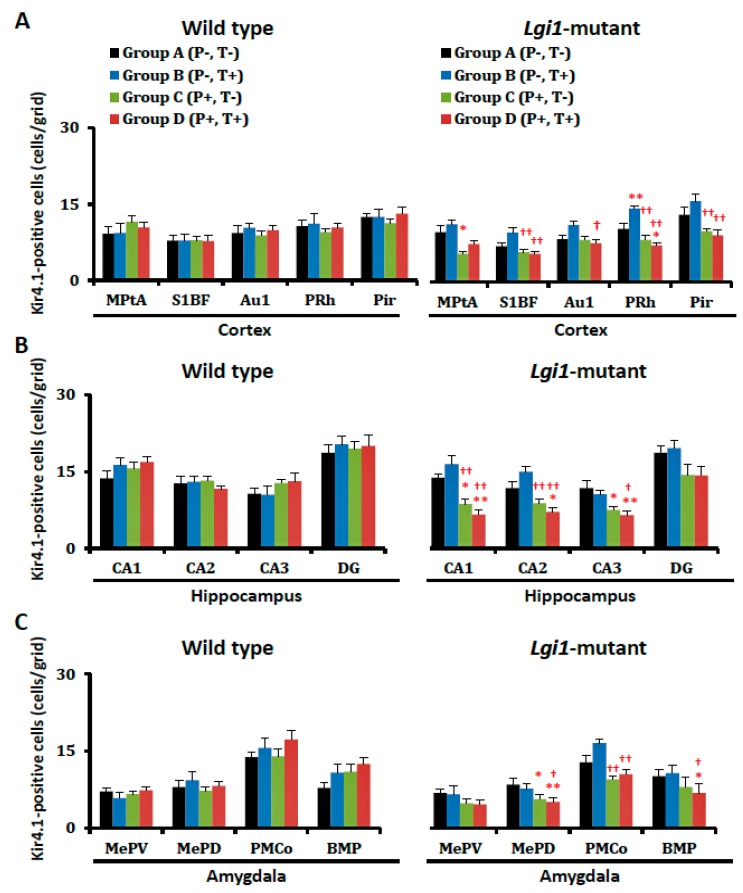
Changes in the number of Kir4.1-immunoreactivity (IR)-positive cells after development of audiogenic epilepsy. Kir4.1-IR-positive cells in each group of wild-type rats (A–D) and *Lgi1* mutant rats are shown in each region of the cortex (**A**), hippocampus (**B**), and amygdala (**C**). Each point represents the mean ± S.E.M. of six to eight animals. * *p* < 0.05, ** *p* < 0.01, significantly different from Group A of wild-type rats and *Lgi1* mutant rats. † *p* < 0.05, †† *p* < 0.01, significantly different from Group B of wild-type rats and *Lgi1* mutant rats.

**Figure 5:**
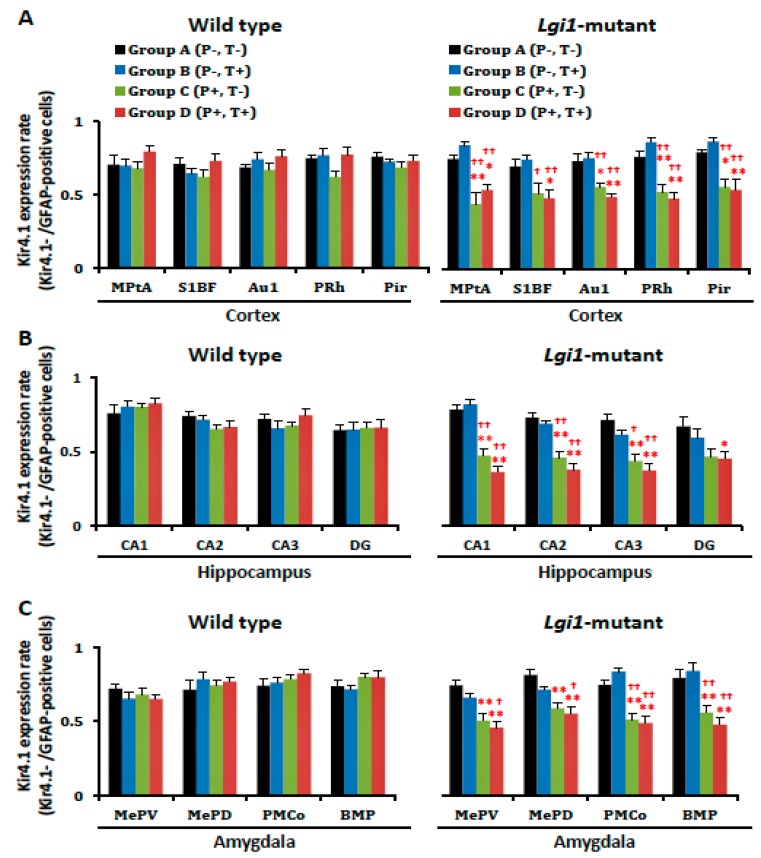
Changes in astrocytic Kir4.1 expression after the development of audiogenic epilepsy. The Kir4.1 expression rate in each group of wild-type rats (A–D) and *Lgi1* mutant rats are shown in each region of the cortex (**A**), hippocampus (**B**), and amygdala (**C**). The Kir4.1 expression rate was expressed as the ration of Kir4.1-immunoreactivity (IR)-positive cells to GFAP-IR-positive cells in each region by staining a pair of successive brain sections with anti-Kir4.1 or anti-GFAP antibodies. Each point represents the mean ± S.E.M. of six to eight animals. * *p* < 0.05, ** *p* < 0.01, significantly different from Group A of wild-type rats and *Lgi1* mutant rats. † *p* < 0.05, †† *p* < 0.01, significantly different from Group B of wild-type rats and *Lgi1* mutant rats.

However, the number of Kir4.1-IR-positive cells was markedly reduced by the priming stimulation in *Lgi1* mutant rats (Group C) (Figure 2C and Figure 4). The Kir4.1 expression rate (ratio of Kir4.1-IR-positive cells versus GFAP-IR-positive cells) in the primed *Lgi1* mutant rats was also significantly decreased by the priming stimulation in the cerebral cortex (Medial parietal association cortex, MPtA; primary auditory cortex, Au1; perirhinal cortex, PRh; and piriform cortex, Pir), hippocampus (CA1, CA2, and CA3), and amygdala (medial amygdaloid nucleus posteroventral part, MePV; medial amygdaloid nucleus posterodorsal part, MePD; posteromedial cortical amygdaloid nucleus, PMCo; and basomedial amygdaloid nucleus posterior part, BMP) (Figure 5). These changes in Kir4.1 expression seemed to be time-dependent, since there was no acute change in Kir4.1 expression in *Lgi1* mutant rats at one day after priming stimulation (P17) (Supplementary Figure S1). Although adiogenic seizures induced by the test stimulation tended to further reduce Kir4.1 expression in some brain regions (e.g., cerebral cortex, hippocampus, and amygdala) of the *Lgi1* mutant rats, these changes between Group C and Group D were not statistically significant (Figure 4 and Figure 5). We also checked the changes in Kir4.1 expression by three-way (i. mutation; ii. priming stimulation; iii. test stimulation) ANOVA, which revealed a statistical significance with the two factors, mutation and priming stimulation, but not with the test stimulation, in most brain regions.

In contrast to *Lgi1* mutant rats, WT rats showed no changes in Kir4.1 expression with or without the priming stimulation at P16 and the test stimulation at eight weeks in all of the examined brain regions (Figure 4 and Figure 5).

### 2.3. Prophylactic Actions of VPA on Seizure Susceptibility in Lgi1 Mutant Rats

**Figure 6:**
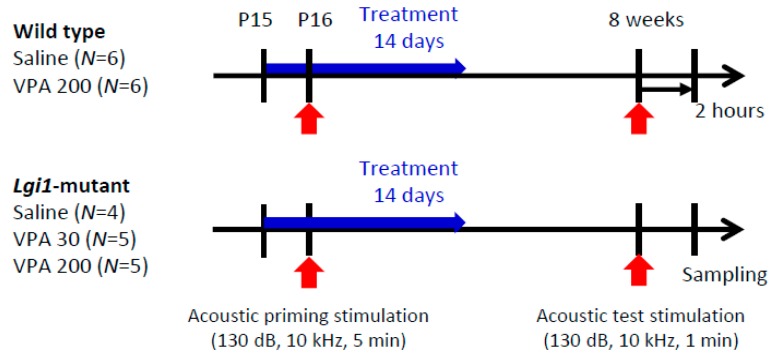
Prophylactic effects of valproic acid (VPA) on audiogenic seizure susceptibility. Animals were intraperitoneally injected with VPA (30 mg/kg and 200 mg/kg) or saline for 14 continuous days from P15 (one day before priming stimulation).

We next examined the prophylactic effects of VPA on the audiogenic seizure susceptibility in *Lgi1* mutant rats. *Lgi1* mutant rats were treated with VPA (30 and 200 mg/kg, i.p.) for two weeks from one day before priming stimulation. Under these conditions, both 30 mg/kg and 200 mg/kg of VPA effectively prevented GTCS induction (Figure 6 and Table 2). Only one out of five *Lgi1* mutant rats (20%) exhibited GTCSs with test stimulation, whereas three out of four *Lgi1* mutant rats (75%) treated with saline showed GTCSs.

### 2.4. Expressional Changes in Astrocytic Kir4.1 with Treatment of VPA

**Figure 7:**
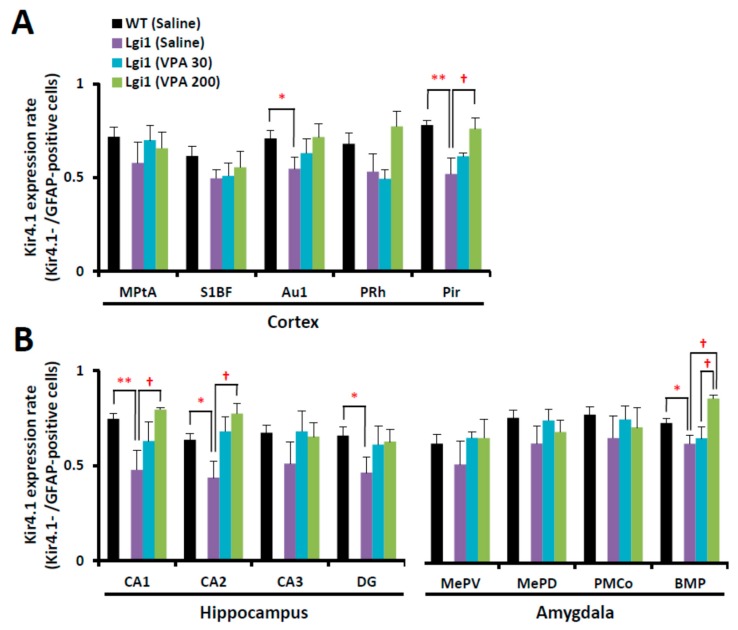
Expression changes in astrocytic Kir4.1 with treatment of valproic acid (VPA). Kir4.1 expression ratios of wild-type rats treated with saline and *Lgi1* mutant rats treated with saline or VPA (30 mg/kg or 200 mg/kg) are shown in each region of the cortex (**A**), hippocampus, and amygdala (**B**). The Kir4.1 expression rate was expressed as the ratio of Kir4.1-IR-positive cells to GFAP-IR-positive cells in each region. Each point represents the mean ± S.E.M. of four to six animals. * *p* < 0.05, ** *p* < 0.01, significantly different from wild-type rats treated with saline. † *p* < 0.05, significantly different from *Lgi1* mutant rats treated with saline.

Similar to the results of prior experiments, the Kir4.1 expression rate significantly decreased in the cerebral cortex (Au1 and Pir), hippocampus (CA1, CA2, and DG), and amygdala (BMP) in the primed *Lgi1* mutant rats treated with saline (Figure 7). However, the prophylactic treatment with VPA (30 mg/kg and 200 mg/kg, i.p.) for two weeks significantly elevated the Kir4.1 expression rate in a dose-related manner in the cerebral cortex (Pir), hippocampus (CA1 and CA2), and amygdala (BMP) (Figure 7).

## 3. Discussion

It has been shown that the hypofunction of Kir4.1 channels in astrocytes was involved in various epileptic disorders including not only epilepsy models in animals [13,14], but also EAST/SeSAME syndrome with *KCNJ10* mutations [10,11,12] and TLE in humans [15,16,17]. Here, we demonstrated for the first time that astrocytic Kir4.1 expression was markedly reduced in the cerebral cortex, hippocampus, and amygdala during the audiogenic epileptogenesis in *Lgi1* mutant rats, which is a rat model of human ADLTE. The brain regions, which showed a down-regulation of Kir4.1 expression, were consistent with the regional distribution of Lgi1 expression [30]. The hippocampus and amygdala were also suggested to be involved in generation of audiogenic seizures in *Fragile X mental retardation 1* (*Fmr1*) knockout mice [31]. It should be noted that the down-regulation of astrocytic Kir4.1 in *Lgi1* mutant rats was obtained without any seizure experience (before applying acoustic test stimulation) after the development of audiogenic epileptogenesis. The present results suggested that the reduced expression of astrocytic Kir4.1 expression is involved in Lgi1-related epileptic disorders. The dysfunction of astrocytic Kir4.1 channels elevates the extracellular levels and K^+^ and glutamate by suppressing the special K^+^ buffering function of astrocytes, which increases the excitability of neurons [4,5,6,7]. In addition, we previously showed that down-regulation of the Kir4.1 channel expression enhanced the expression of BDNF, which is a key modulator of epileptogenesis, in astrocytes [18,19]. Knockdown of the BDNF gene [32,33,34,35] or inhibition of the BDNF receptor TrkB [36,37] has been shown to suppress seizure development. Thus, it seems possible that the down-regulation of astrocytic Kir4.1 channels participates in the development of epilepsy in *Lgi1* mutant rats.

Epileptogenesis generally shows a time lag until the development of seizures following trigger events (e.g., stroke, traumatic brain injury, and status epilepticus) [38]. In audiogenic epileptogenesis caused by *Lgi1* mutation, astrocytic Kir4.1 expression also required time to be down-regulated. Although the mechanisms underlying the down-regulation of Kir4.1 expression by *Lgi1* mutation are still uncertain, it has been recently demonstrated that Kir4.1 channel expression in astrocytes was down-regulated by increased glutamate signaling [39]. Since previous studies have suggested that the synaptic release of glutamate is increased by *Lgi1* mutation [40,41], this might be involved in the down-regulation of astrocytic Kir4.1 expression in *Lgi1* mutant rats. In addition, Lgi1 is known to regulate the expression and/or clustering of potassium channels (e.g., Kv1.1 channels) through the interaction with the membrane-anchored proteins (ADAM22 and ADAM23) [23,26]. Thus, disruptions of the Lgi1–ADAM22/23 interaction in *Lgi1* mutant rats might also influence the Kir4.1 channel expression in astrocytes. Further studies are required to clarify the mechanisms underlying the interaction of Lgi1 with Kir4.1 channels in the *Lgi1*-related epileptogenesis.

VPA is widely used as an antiepileptic drug that is effective for both generalized and partial seizures. It enhances GABAergic neurotransmission by inhibiting the GABA metabolic enzyme, GABA transaminase [19,42]. In addition, VPA also blocks voltage-gated sodium channels and T-type calcium channels. These actions are considered to cause neuronal inhibition and contribute to its anticonvulsive activity. Nonetheless, several studies have suggested that VPA shows prophylactic effects on seizure generation and might have the potential to prevent epileptogenesis [43,44,45]. In consistent with this possibility, we have previously shown that repeated treatment of animals with VPA enhanced astrocytic Kir4.1 expression in the cerebral cortex and limbic regions (hippocampus and amygdala), which seems to inhibit epileptogenesis via facilitating the spatial potassium buffering function of astrocytes. In the present study, we confirmed that VPA could alleviate the epileptogenesis in *Lgi1* mutant rats with elevating astrocytic Kir4.1 expression. Our results suggest that VPA can prevent or alleviate Lgi1-related epileptogenesis by normalizing the down-regulation of the astrocytic Kir4.1 channel.

In conclusion, we analyzed the astrocytic Kir4.1 expression during audiogenic epileptogenesis in *Lgi1* mutant rats. Kir4.1 expression in astrocytes was significantly decreased by the postnatal priming stimulation in the cerebral cortex, hippocampus, and amygdala. In addition, prophylactic treatments with VPA effectively alleviated audiogenic seizure susceptibility and prevented the reduced expression of astrocytic Kir4.1 channels in *Lgi1* mutant rats. The present study suggests that the down-regulation of Kir4.1 channels in astrocytes is involved in audiogenic seizure generation caused by *Lgi1* mutation. Moreover, VPA seemed to have a prophylactic effect on *Lgi1*-related epileptogenesis. However, this study evaluated the Kir4.1 levels solely by counting IR-positive cells, but not by calculating the IR area [46], so that compensatory changes represented by increased Kir4.1-IR in the remaining positive cells could not be excluded. Further analyses of Kir4.1 expression at cellular and subcellular levels are necessary to delineate the mechanisms underlying the Kir4.1 down-regulation by *Lgi1* mutation and its relationship to epileptogenesis.

## 4. Materials and Methods

### 4.1. Animals

F344-*Lgi1^m1Kyo^* rats (NBRP Rat No. 0656), carrying a heterozygous missense mutation (L385R/+) generated by *N*-ethyl-*N*-nitrosourea (ENU) mutagenesis [27], were obtained from the National BioResource Project-Rat (NBRP-Rat, http://www.anim.med.kyoto-u.ac.jp). The housing conditions of the rats and animal care methods complied with the Guide for the Care and Use of Laboratory Animals of the Ministry of Education, Science, Sports and Culture of Japan. The experimental procedures used were approved by the Animal Research Committee of Kyoto University (Med Kyo 14560, 6 November 2014) and Osaka University of Pharmaceutical Sciences (No.15, 30 March 2015). Only male rats were used for phenotypic analyses.

### 4.2. Genotyping

F344-*Lgi1^m1Kyo^* rats were mated with their wild-type littermates. The blood samples of rat pups at P15 were used for genotyping using a PCR restriction fragment length polymorphism (RFLP) technique. Briefly, exon 8 of *Lgi1*, including the mutation site, was amplified by PCR with primers (forward: 5′-CCACACATCTAATGTCTCATCTGTT-3′, reverse: 5′-AGGATCAAATGAGGTGTTCTGAG-3′) using the Ampdirect Plus PCR buffer (Shimadzu, Kyoto, Japan). The PCR products were digested with *Xsp* I (Takara Bio, Shiga, Japan); then, fragments (mutant allele: 399 bp, wild allele: 350 bp) were separated by conventional agarose gel electrophoresis.

### 4.3. Audiogenic Seizure Induction

Animals divided into four groups (Group A–D) were individually exposed to auditory stimulation using a previously reported method [27,28] (Figure 1). Priming stimulation was performed in P16 rats with a sound stimulus (130 dB, 10 kHz, five minutes). Test stimulation was performed with a sound stimulus (130 dB, 10 kHz, one minute) at eight weeks. Within two hours after seizure induction or a sham procedure, the animals were deeply anesthetized with pentobarbital (80 mg/kg, i.p.) that was transcardially perfused with ice-cold phosphate-buffered saline (PBS), and then with 4% paraformaldehyde solution. The brains were removed from the skull and placed in fresh fixative for 24 h.

### 4.4. Immunofluorescence Staining

Immunofluorescence double staining of Kir4.1 with GFAP (a specific marker for astrocytes) was performed as published previously [14,20,29]. Briefly, fixed brain samples were embedded in paraffin and cut into four-μm thick sections. Sections were autoclaved for 20 min to retrieve the antigen, and blocked with 1% bovine serum albumin (BSA) for 30 min. The sections were incubated with primary antibodies for GFAP (mouse monoclonal, 1:50; Progen, Heidelberg, Germany) and Kir4.1 (rabbit polyclonal, 1:100; Alomone Labs, Jerusalem, Israel) at 4 °C overnight. Subsequently, secondary antibodies of tetramethylrhodamine-5- (and 6)-isothiocyanate (TRITC; red fluorescence) goat anti-mouse (1:50; Sigma-Aldrich, St. Louis, MO, USA), or fluorescein isothiocyanate (FITC; green fluorescence) goat anti-rabbit (1:50; Sigma-Aldrich) were respectively used for visualization. Immunofluorescence images were obtained with a confocal laser scanning microscope (Carl Zeiss Japan, LSM 700 ZEN, Tokyo, Japan).

### 4.5. Immunohistochemical Analysis

Kir4.1 and GFAP expression in each brain region was analyzed with immunohistochemical staining using the ABC method, as published previously [14,20,29]. After blocking, sections were incubated with rabbit anti-Kir4.1 antibodies (1:100; Alomone Labs) or mouse anti-GFAP antibodies (1:100; Progen) at 4 °C overnight. Thereafter, they were incubated with biotinylated goat anti-rabbit IgG antibodies (1:400, Vector Laboratories, Burlingame, CA, USA) or biotinylated goat anti-mouse IgG antibodies (1:400, Sigma-Aldrich) for 60 min and with avidin-biotinylated horseradish peroxidase complexes (Vectastain ABC Kit, Vector Laboratories) for an additional 60 min. Kir4.1-IR and GFAP-IR were visualized by the diaminobenzidine-nickel staining method.

Kir4.1 and GFAP expression were quantified by counting the number of Kir4.1-IR-positive or GFAP-IR-positive cells in a 350 × 350 µm^2^ grid laid over each region of the brain, as shown in Figure 2B, which included the following regions: medial parietal association cortex (MPtA), primary somatosensory cortex barrel field (S1BF), primary auditory cortex (Au1), perirhinal cortex (PRh), piriform cortex (Pir), hippocampal CA1, CA2, CA3, and dentate gyrus (DG), medial amygdaloid nucleus posteroventral part (MePV), medial amygdaloid nucleus posterodorsal part (MePD), posteromedial cortical amygdaloid nucleus (PMCo), basomedial amygdaloid nucleus posterior part (BMP), basolateral amygdaloid nucleus posterior part (BLP), lateral amygdaloid nucleus ventromedial part (LaVM), lateral habenula (LHb), ventromedial thalamus (VM), posterior hypothalamus (PH), dorsomedial hypothalamic nucleus, and dorsal part (DMD). The Kir4.1 expression was expressed as a percentage of the number of Kir4.1-IR-positive cells relative to that of GFAP-IR-positive cells obtained from successive slices of the same animals.

### 4.6. Drug Treatments

To determine whether an antiepileptic agent modified the pathophysiological alterations in Kir4.1 channels in Lgi1-related epileptogenesis, we studied the prophylactic actions of VPA in *Lgi1* mutant rats, with a focus on its regulation of Kir4.1 channel expression. From one day before priming stimulation, animals were intraperitoneally injected with VPA (30 mg/kg and 200 mg/kg) (Sigma-Aldrich) or saline for 14 continuous days (Figure 6). Then, the animals were subjected to the audiogenic seizure test four weeks after the last VPA treatment, and brain samples were obtained as described previously.

### 4.7. Statistical Analysis

All of the data were expressed as the mean ± S.E.M. Comparisons between two groups (expressional changes of Kir4.1 determined by immunohistochemical analysis in each region) were performed by a parametric Student’s *t*-test. Statistical significance of differences among multiple groups (expressional changes of Kir4.1 or GFAP determined by immunohistochemical analysis in each region) was determined by one-way ANOVA followed by Tukey’s post hoc test. A *p*-value of less than 0.05 was considered statistically significant.

## Figures and Tables

**Table 1:** The responses to acoustic test stimulation at eight weeks in wild-type rats and *Lgi1* mutant rats. WR: wild running; GTCS: generalized tonic–clonic seizure.

Genotype	Acoustic Stimulation		Responses to Test Stimulation (No. of Animals)			
	Priming	Test	Total	None	WR	WR+ GTCS
Group B Wild type	-	+	6	6	0	0
Group B *Lgi1*-mutant	-	+	7	7	0	0
Group D Wild type	+	+	8	0	8	0
Group D *Lgi1*-mutant	+	+	8	0	1	7 (88%)

**Table 2:** The responses to acoustic test stimulation at eight weeks in wild-type rats and *Lgi1* mutant rats treated with VPA. WR: wild running; GTCS: generalized tonic–clonic seizure.

Genotype	Drug (mg/kg)	Responses to Test Stimulation (No. of Animals)			
		Total	None	WR	WR + GTCS
Wild type	Saline	6	0	6	0
Wild type	VPA 200	6	0	4	0
*Lgi1*-mutant	Saline	4	0	1	3 (75%)
*Lgi1*-mutant	VPA 30	5	0	4	1 (20%)
*Lgi1*-mutant	VPA 200	5	0	4	1 (20%)

## Supplementary Materials

Supplementary materials can be found at https://www.mdpi.com/1422-0067/20/5/1013/s1.
