# Supplementary material for: Down-Regulation of Astrocytic Kir4.1 Channels during the Audiogenic Epileptogenesis in Leucine-Rich Glioma-Inactivated 1 (Lgi1) Mutant Rats
Source: Int J Mol Sci. 2019 Feb 26;20(5):1013. doi: 10.3390/ijms20051013 (PMC6429235; doi:10.3390/ijms20051013)
Supplement: Supplementary file 1 [file ijms-20-01013-s001.pdf]

Down-Regulation of Astrocytic Kir4.1 Channels during the Audiogenic Epileptogenesis in *Leucine-Rich Glioma-Inactivated 1 (Lgi1)* Mutant Rats

Supplementary materials

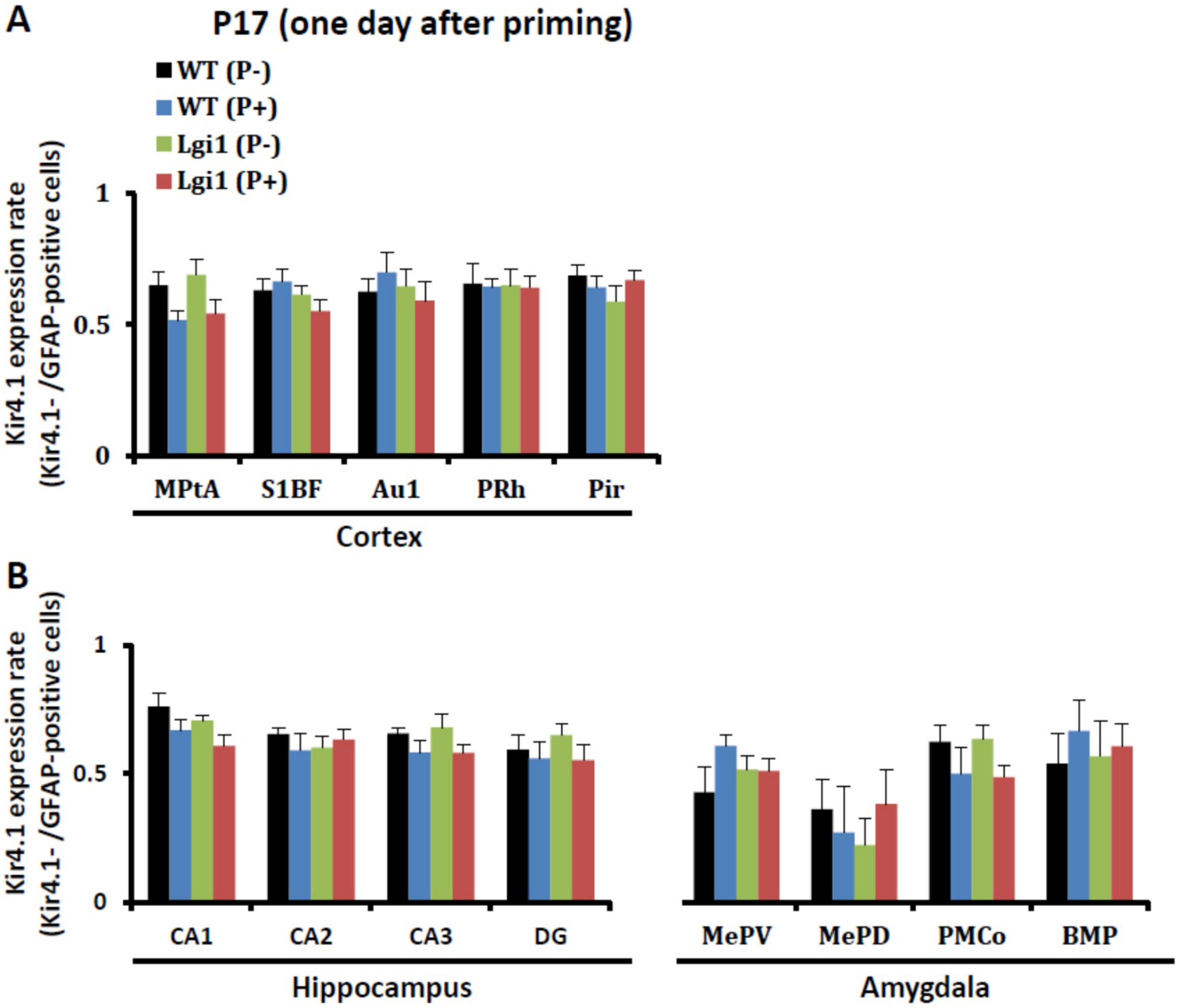

Supplementary Figure S1. Expression changes in astrocytic Kir4.1 at one day after priming stimulation (P17). Kir4.1 expression ratios in each region of the cortex (A), hippocampus, and amygdala (B) are shown in primed or not primed wild-type (WT) rats and primed or not primed *Lgi1* mutant rats. The Kir4.1 expression rate was expressed as the ration of Kir4.1-immunoreactivity (IR)-positive cells to GFAP-IR-positive cells in each region. Each point represents the mean ± S.E.M. of four to eight animals.
